# Hearing thresholds at high frequency in patients with cystic fibrosis: a systematic review^☆^

**DOI:** 10.1016/j.bjorl.2016.10.006

**Published:** 2016-11-09

**Authors:** Debora T.M. Caumo, Lúcia B. Geyer, Adriane R. Teixeira, Sérgio S.M. Barreto

**Affiliations:** a Universidade Federal do Rio Grande do Sul (UFRGS), Faculdade de Medicina (Famed), Saúde da Criança e do Adolescente, Porto Alegre, RS, Brazil; b Hospital de Clínicas de Porto Alegre (HCPA), Serviço de Otorrinolaringologia, Porto Alegre, RS, Brazil; c Universidade Federal do Rio Grande do Sul (UFRGS), Instituto de Psicologia, Departamento de Saúde e Comunicação Humana, Porto Alegre, RS, Brazil; d Universidade Federal do Rio Grande do Sul (UFRGS), Faculdade de Medicina (Famed), Departamento de Medicina Interna, Porto Alegre, RS, Brazil

**Keywords:** Cystic fibrosis, Audiometry, Ototoxicity, Hearing loss, Fibrose cística, Audiometria, Ototoxicidade, Perda auditiva

## Abstract

**Introduction:**

High-frequency audiometry may contribute to the early detection of hearing loss caused by ototoxic medications. Many ototoxic drugs are widely used in the treatment of patients with cystic fibrosis. Early detection of hearing loss should allow known harmful drugs to be identified before the damage affects speech frequencies. The damage caused by ototoxicity is irreversible, resulting in important social and psychological consequences. In children, hearing loss, even when restricted to high frequencies, can affect the development of language.

**Objective:**

To investigate the efficacy and effectiveness of hearing monitoring through high-frequency audiometry in pediatric patients with cystic fibrosis.

**Methods:**

Electronic databases PubMed, MedLine, Web of Science and LILACS were searched, from January to November 2015. The selected studies included those in which high-frequency audiometry was performed in patients with cystic fibrosis, undergoing treatment with ototoxic drugs and published in Portuguese, English and Spanish. The GRADE system was chosen for the evaluation of the methodological quality of the articles.

**Results:**

During the search process carried out from January 2015 to November 2015, 512 publications were identified, of which 250 were found in PubMed, 118 in MedLine, 142 in Web of Science and 2 in LILACS. Of these, nine articles were selected.

**Conclusion:**

The incidence of hearing loss was identified at high frequencies in cystic fibrosis patients without hearing complaints. It is assumed that high-frequency audiometry can be an early diagnostic method to be recommended for hearing investigation of patients at risk of ototoxicity.

## Resumo

**Introdução:**

A audiometria de altas frequências pode contribuir para a detecção precoce de alterações auditivas causadas por medicações ototóxicas. No tratamento dos pacientes com fibrose cística, existem muitos fármacos ototóxicos amplamente utilizados. A detecção precoce de alterações auditivas deve permitir que sejam identificadas antes que o dano atinja as frequências da fala. A lesão causada pela ototoxicidade é irreversível, traz importantes consequências sociais e psicológicas. Nas crianças, a perda auditiva, mesmo restrita às altas frequências, pode afetar o desenvolvimento da linguagem.

**Objetivo:**

Investigar a eficácia e a efetividade do monitoramento da audição por meio da audiometria de altas frequências em pacientes pediátricos com fibrose cística.

**Método:**

Foram consultadas as bases de dados eletrônicas PubMed, Medline, *Web of Science* e Lilacs, de janeiro a novembro de 2015. Foram selecionados os estudos em que foi feita audiometria de altas frequências em pacientes com fibrose cística em tratamento com medicamentos ototóxicos e publicados em português, inglês e espanhol. Para a avaliação da qualidade metodológica dos artigos optou-se pela utilização do Sistema Grade.

**Resultados:**

No processo de busca feito de janeiro de 2015 a novembro de 2015 foram encontradas 512 publicações, sendo 250 na PubMed, 118 na Medline, 142 na *Web of Science* e dois na Lilacs. Desses, foram selecionados nove artigos.

**Conclusão:**

Identificou-se a ocorrência de perda auditiva em altas frequências em pacientes com fibrose cística sem queixas auditivas. Admite-se que audiometria em altas frequências possa ser um método de diagnóstico precoce a ser recomendado para investigação auditiva de pacientes em risco de ototoxicidade.

## Introduction

Cystic fibrosis (CF), also called mucoviscidosis, is caused by mutations in a gene located on the long arm of chromosome 7 (7q31). This gene encodes the cystic fibrosis transmembrane conductance regulator (CFTR), which functions as a chloride channel, performing and controlling ion transportation through the cell membrane.^1^

Individuals with CF have chloride secretion failure in the respiratory epithelium, which results in excessive sodium absorption, resulting in increased influx of water into cells and therefore elevating mucus viscosity. The mucus becomes approximately 30–60 times thicker than normal. It does not directly affect ciliary beat, but ciliary action becomes ineffective in the clearance of such highly viscous substance, causing stasis, predisposing to ostial obstruction and increased bacterial colonization.^2^, ^3^ This can lead to recurrent pulmonary infections, chronic obstructive pulmonary disease, sinusitis, nasal polyps, gastrointestinal malabsorption secondary to pancreatic failure, meconium ileus, rectal prolapse and infertility due to obstruction of the vas deferens.^2^

The incidence of CF varies according to ethnicity, occurring more commonly in Caucasians. Being an autosomal recessive disease, when each parent has the gene for CF, the probability of the birth of a child with the disease is 25%. In accessing ethnic groups, the incidence of CF varies from 1/2000 to 1/5000 live births among Caucasians in Europe, the United States and Canada, 1/15,000 in African-Americans, and 1/40,000 in Finland.^4^, ^5^

In Brazil, the estimated incidence for the southern region is closer to that of the Central European Caucasian population, whereas for the other regions, it decreases to approximately 1/10,000 live births.^4^ The prevalence of the mutation in the CFTR gene (ΔF508), in the pediatric population of patients with CF born in Porto Alegre is 60%, with an estimated incidence of 10 cases a year in the city.^6^

The diagnosis of CF is essentially clinical, although neonatal screening has allowed the diagnosis of cases even before symptom onset. The diagnosis can be made through the identification of two mutations in the CF gene, two abnormal results of the sweat test, or the presence of at least one of the following symptoms: obstructive/suppurative lung disease or chronic sinus disease, chronic exocrine pancreatic failure or family history of CF.^2^, ^4^ For patients in whom the chloride concentration in sweat is normal or borderline and in which mutations in the two CF genes are not identified, the nasal potential difference (NPD) measurement can be used as evidence of CFTR dysfunction. Clinical decision-making will continue to be essential for patients that have “atypical” or typical symptoms, but have no conclusive evidence of CFTR dysfunction.^7^

When treating patients with CF, many ototoxic drugs are widely used, including aminoglycosides (AGs), which may cause hearing loss. Some AGs are classified as more vestibulotoxic (affecting the vestibular system), whereas others are more cochleotoxic (affecting the cochlea). Therefore, the monitoring of these patients’ hearing is recommended for early detection of signs, even asymptomatic ones, of deterioration and damage to the auditory system.^8^

Ototoxic drugs can damage the organ of Corti directly, first degenerating the outer hair cells, starting with the spirals at the base of the cochlea and spreading toward its apex. The cell degeneration may extend to the inner hair cells, starting in the cochlea apex, if exposure to the ototoxic agent persists.^9^

Ototoxicity has played an important role, especially in younger children, since it is generally considered irreversible and results in severe future harm.^10^ Audiometric tests that assess high frequencies are especially used to detect ototoxicity-related sensorineural hearing loss.^11^

High-frequency audiometry (HFA) is mentioned in the international literature as sensitive for the early detection of hearing loss caused by ototoxic medications. Although there is no criterion for result interpretation, many researchers have stated that hearing monitoring is essential to prevent a degeneration process from occurring in the basal spiral of the cochlea.^12^, ^13^, ^14^, ^15^

HFA is considered a subjective hearing assessment, carried out in a soundproof booth, using headphones that are specially calibrated to emit high-pitched sounds of 8000–16,000 Hz.^15^ This test contributes to a better understanding of cochlear lesion at higher frequencies, which cannot be obtained with conventional audiometry, of which frequency range goes only up to 8000 Hz.

Hearing monitoring should allow lesion identification before the damage reaches speech frequencies and thus threaten their understanding. According to previous studies, it is well established that hearing loss caused by ototoxic drugs begins at the base of the cochlea, affecting primarily the higher frequencies.^16^

HFA seems to identify asymptomatic hearing damage in CF patients and may contribute to the early detection of hearing loss.^17^, ^18^ The search for scientific evidence in this context becomes necessary, as these patients usually have access to conventional audiometry, but could benefit even further from the hearing evaluation at higher frequencies. The objective of this research is to evaluate the accuracy of the HFA in identifying hearing loss in pediatric patients with CF.

## Methods

A systematic review was carried out in the PubMed, MedLine, Web of Science and LILACS databases. There was no restriction regarding the year of publication. The following descriptors from DeCS (Health Sciences Descriptors) were used: audiometry, hearing loss, cystic fibrosis, aminoglycosides and abnormalities, drug-induced, as well as their corresponding terms in Portuguese.

The evaluation of the titles and abstracts identified in the initial search was carried out independently by two researchers, strictly following the inclusion and exclusion criteria previously defined in the research protocol. Studies were included when the title, subject and/or keywords were consistent with the purpose of this study carried out in children and adolescents with cystic fibrosis receiving treatment with ototoxic medication. The interventions should include HFA, which is the measurement of the outcome of interest (hearing thresholds at high frequencies). Articles written in Portuguese, Spanish and English were considered.

To evaluate the methodological quality of the articles, we chose to use the GRADE (Grades of recommendation, assessment, development, and evaluation) system. This system provides, in addition to criteria for the degree of evidence, a definition for the quality of evidence. This system aims to be clear and explicit, considering the study design, performance, consistency, as well as direct and linear direction when assessing quality of evidence for each important outcome/consequence.^19^, ^20^ The quality of evidence according to the GRADE system is shown as follows^21^:

• **High (A)**: Consistent, with evidence from randomized controlled trials or meta-analyses without significant limitations or with exceptionally strong evidence from observational studies. It is very unlikely that additional research will change the confidence in the estimated effects.
• **Moderate (B)**: Evidence from randomized controlled trials with significant limitations (inconsistent results, methodological flaws, inaccuracies, indirect results). Additional research is likely to have an impact on the confidence of the estimate of effect and may change this estimate.
• **Low (C)**: Evidence from at least one important result of observational studies, series of cases or randomized controlled trials with serious flaws or indirect evidence. It is very likely that further research will have a significant impact on the confidence of the effect estimate and is likely to change the estimate.
• **Very low (C)**: Any effect estimate is uncertain.

The evidence that constitutes systematic reviews are not directly indicated as recommendations, but constitute tools that support the final recommendations of guidelines. The GRADE system considers as strong the recommendation of a diagnostic test when the benefits clearly outweigh the risks and “burden of disease” or vice versa. When the benefits are limited, showing risks and “burden of disease”, the GRADE system classifies it as weak recommendation.^22^

## Results

**Figure 1:**
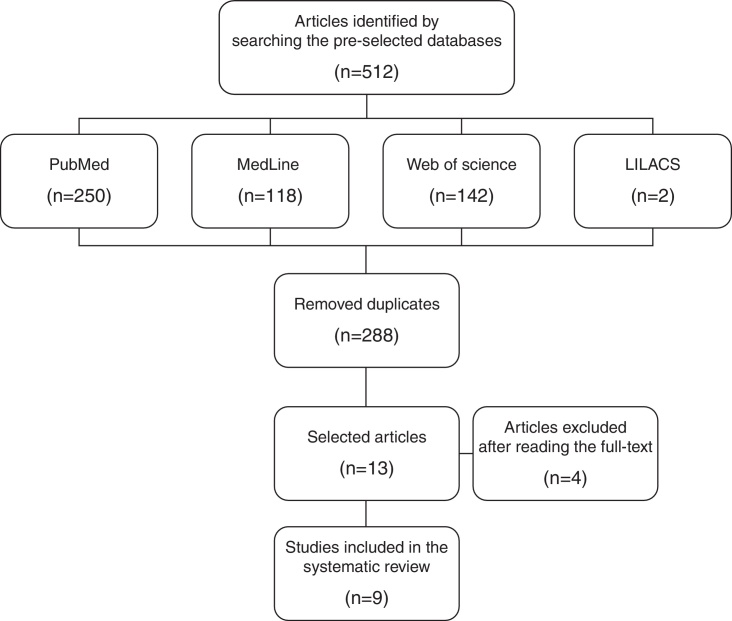
Flowchart of the search and selection process of studies on auditory thresholds in pediatric patients with cystic fibrosis treated with ototoxic medications.

During the search process, carried out from January to November 2015, 512 publications were identified (250 in PubMed, 118 in MedLine, 142 in Web of Science and 2 in LILACS). After the exclusion of repeated articles in the surveyed databases, a total of 224 articles were obtained. After reading the titles and abstracts, 211 articles were excluded, as they did not assess the outcome or exposure of interest. The remaining 13 publications were reviewed by reading and assessing the full text. At this stage, four publications were excluded, as they did not assess the outcome of interest. Thus, the search strategy identified nine articles that met the inclusion criteria for this review according to the study selection flowchart (Fig. 1). The nine publications selected after the search strategy are shown in Table 1 and the qualification of evidence according to the GRADE system is shown in Table 2.

**Table 1:** Main characteristics of the studies.

Title/author/year	Design	Sample	Intervention	Results	Conclusion
Absence of Cochleotoxicity measured by standard and high-frequency pure tone audiometry in a trial of once- vs. three times-daily tobramycin in cystic fibrosis patients (Mulheran et al., 2006)	Experimental study: randomized controlled trial. Web of Science, PubMed – indexed for MedLine	244 patients with Cystic Fibrosis (CF) were included, of which 219 (125 children and 94 adults) underwent audiometry. Results were obtained for 168 of the total number of patients who completed treatment.	Pure tone audiometry was performed throughout the frequency range of 0.25–8 kHz. High-frequency audiometry of 10–16 kHz. Audiometric tests were performed at the beginning of the treatment with tobramycin, at the end of a 14-day treatment, and 6–8 weeks later.	No significant differences were detected in the auditory thresholds between treatment regimens. There was loss of hearing thresholds at high frequencies in both groups of treatment regimens.	No cumulative cochleotoxicity risk was demonstrated in patients with CF due to repeated therapy with aminoglycosides, requiring a better characterization.
Aminoglycoside antibiotics cochleotoxicity in pediatric Cystic Fibrosis (CF) patients (Al-Malky et al., 2011)	Observational cohort study: Web of Science, PubMed–indexed for MedLine	45 children with cystic fibrosis (CF). 39/45 participants had received aminoglycosides (AGs), through intravenous (IV) route, 23 of them having received it repeatedly every 3 months.	Audiometric tests and distortion-product otoacoustic emissions (DPOAE) were performed during routine visits to the CF outpatient clinic or when patients were hospitalized. Participants were grouped according to their prior history of AG exposure intravenously.	In the high-exposure group, 8 (21%) had clear signs of ototoxicity; thresholds at 8–20 kHz were elevated by ∼50 dB. DPOAE amplitudes were >10 dB lower in f2 3.2–6.3 kHz. The remaining 31/39 (79%) patients exposed to treatment with AGs maintained normal hearing threshold.	A significant number of children with CF had ototoxicity due to repeated courses of treatment with AGs intravenously. It is recommended to perform auditory tests in all CF patients with high exposure to AG. The occurrence of hearing loss (HL) was associated with high exposure; however, high exposure only resulted in HL in a minority of patients. Genetic analysis may help explain the dichotomy found in response to AGs.
Aminoglycoside ototoxicity in cystic fibrosis. Evaluation by high-frequency audiometry (McRorie, Bosso, Randolph, 1989)	Observational study: paired case-control. Web of Science, PubMed – indexed for MedLine	22 patients with Cystic Fibrosis (CF) treated with aminoglycosides (AGs). 13 of them were paired and compared to 38 individuals without CF, who had never received treatment with AGs.	Audiometric tests were performed to measure hearing thresholds of 250–20,000 Hz.	In patients with CF that were treated with AGs (younger than 20 years), there were statistically significant differences in frequencies > 16 kHz. CF patients who were treated with AG older than 20 years had elevated thresholds at all tested frequencies. CF patients who were not treated with AG did not differ statistically.	The high-frequency audiometry can be a useful measure of the increase in hearing thresholds preceding the noticeable loss of hearing acuity in CF patients who are treated with long-term AGs.
Assessment of ultra-high frequency audiometry use in patients receiving ototoxic drugs (Weigert et al., 2013)	Observational, cross-sectional study with unpaired control group. LILACS	69 individuals aged 7–20 years of age. 35 patients regularly submitted to therapy with potentially ototoxic medication that were part of the study group (SG) and 34 healthy subjects that were the control group (CG).	Conventional audiometry (250–8000 Hz) and audiometry at ultra-high frequencies (9000–16,000 Hz) were performed.	There was one altered case in the conventional audiometry (2.9%) and 6 altered cases in the audiometry at ultra-high frequencies (17.1%) (*p* = 0.063). The SG had statistically higher thresholds in relation to the CG in the conventional audiometry at the frequencies of 2 kHz and 8 kHz and, in the audiometry with ultra-high frequencies, at 10 kHz (*p* = 0.004) and 16 kHz (*p* < 0.001).	The study suggested that the audiometry in ultra-high frequencies is useful for hearing monitoring in patients at risk for ototoxicity.
Cumulative and acute toxicity of repeated high-dose tobramycin treatment in cystic fibrosis (Pedersen et al., 1987)	Observational, cross-sectional study. Web of Science, PubMed indexed for MedLine	46 patients with Cystic Fibrosis (CF), mean age of 15.7 years and chronic bronchopulmonary infection by *Pseudomonas aeruginosa*.	Patients were studied before and at the end of a two-week course of treatment with tobramycin (10–20 mg/kg per day) to discriminate between acute and chronic toxicity using audiometry and electronystagmography tests.	Two patients (5%) had hearing loss at high frequency (above 8 kHz), but only one related to tobramycin. No chronic vestibular toxicity was observed. After 2 weeks of treatment, 32% had a slightly reduced hearing threshold (15–30 dB) at two or more high frequencies and 28% had a decrease in the superior vestibular response at 25% of the initial value, but remained within normal limits.	The acute and chronic toxicity during treatment with high doses of tobramycin in CF patients seems to be very mild.
Hearing loss in cystic fibrosis (Martins et al., 2010)	Observational, cross-sectional, descriptive study. Web of Science, PubMed indexed for MedLine	120 patients with Cystic Fibrosis (CF) aged 5 months to 18 years.	The study was carried out through questionnaires, audiometric testing and analysis of distortion-product otoacoustic emissions (DPOAE). The evaluation of prior use of aminoglycoside antibiotics (AGs) was performed by collecting data from medical records.	Audiometric tests showed a 4–11% prevalence of sensorineural hearing loss. There was no statistically significant association between the use of AGs and hearing loss (*p* = 0.48).	Analysis of audiometric tests and DPOAE showed that there was a high prevalence of hearing loss, which makes CF patients a group of high risk that requires periodic hearing assessment. The comparison of the groups with and without use of AGs showed no statistically significant difference, suggesting that the use of AGs is not the only causal factor for hearing loss in CF.
High-frequency audiometry reveals high prevalence of aminoglycoside ototoxicity in children with cystic fibrosis (Al-Malky et al., 2015)	Observational, cross-sectional, prospective study. Web of Science, PubMed – in process	70 children with Cystic Fibrosis (CF). Mean age of 10.7 years.	Hearing assessment was performed in children with CF using standard pure tone audiometry (PTA) and high frequency audiometry (HFA), as well as distortion-product otoacoustic emissions (DPOAE).	Of the 63 children who received intravenous (IV) AGs, 15 (24%) had ototoxicity detected by HFA and DPOAE. PTA detected ototoxicity in 5 children (only at the frequency of 8 kHz, with hearing loss >20 dB HL) and 13 children with some non-significant change. A hearing loss of 25–85 dB HL at all high frequencies and a significant decrease in DPOAE amplitude at frequencies of 4–8 kHz were detected. There was a statistically significant difference (*p* < 0.05) in the hearing threshold between the groups at high frequencies (8–16 kHz).	Children with CF who received at least 10 AG courses through IV route had a higher risk of ototoxicity. HFA identified 2 children with ototoxicity more than PTA. Depending on the available facilities, HFA must be the test of choice for the detection of ototoxicity in children with CF who received treatment with AGs.
High frequency hearing thresholds and product distortion otoacoustic emissions in cystic fibrosis patients (Geyer, Barreto, Weigert, Teixeira, 2015)	Observational cross-sectional, retrospective and prospective study with a control group. MedLine PubMed-in process	Children and adolescents from adult and pediatric Cystic Fibrosis (CF) outpatient clinics. The sample consisted of 75 individuals, 39 in the Study Group (SG) and 36 in the control group (CG).	Existing data were used, from a database created earlier. Further tests were added to this database, by conducting pure tone audiometry (PTA), High-Frequency Audiometry (HFA) and distortion product otoacoustic emissions (DPOAE) in more patients from the Cystic Fibrosis Outpatient Clinic (SG) and Control Group (CG).	The SG had significantly higher thresholds at 250, 1000, 8000, 9000, 10,000, 12,500 and 16,000 Hz; (*p* = 0.004). There was a significant association between changes in hearing thresholds in the HFA with the number of courses of aminoglycosides (AGs) that were carried out (*p* = 0.005). 83% of patients who had more than 10 courses of AGs had hearing loss in the HFA.	A significant number of CF patients who received repeated AGs courses showed changes in the HFA and DPOAE. Receiving 10 or more AG courses was associated with changes in the HFA.
Occurrence and risk of cochleotoxicity in cystic fibrosis patients receiving repeated high-dose aminoglycoside therapy (Mulheran et al., 2001)	Observational cross-sectional, retrospective study with a control group. Web of Science, PubMed – indexed for MedLine	70 patients with CF subdivided into groups; 27 pediatric patients (10–18 years) and 43 adults (19–37 years). Results were compared with the results of 91 individuals from the control group.	Standard pure tone audiometry (0.25–8 kHz) and high-frequency audiometry (10–16 kHz) were performed.	Of 70 patients with CF, 12 (one pediatric patient) had hearing loss considered to be caused by repeated exposure to aminoglycosides (AGs). There was a nonlinear association between the therapy courses received and the incidence of hearing loss. Loss severity does not seem to be associated with the number of courses received. Preliminary risk estimates of hearing loss per AG course were less than 2%.	After comparison with previous clinical studies and experimental work, these findings suggest that the incidence of cochleotoxicity in CF patients is considerably lower than expected, suggesting that the CF condition can confer protection against cochleotoxicity.

**Table 2:** GRADE System for quality of evidence.

Author/year/design	Class A	Class B	Class C
Geyer LB, Menna Barreto SS, Weigert LL, Teixeira AR, 2015 (Observational, cross-sectional study, with paired control group).			X
Al-Malky G et al., 2015 (Observational, cross-sectional study).			X
Weigert LL, et al., 2013 (Observational, cross-sectional study, with non-paired control group).			X
Al-Malky G, et al., 2011 (Cohort study).		X	
Mulheran M, et al., 2006 (Randomized clinical trial).	X		
Martins LMN, et al., 2010 (Observational, cross-sectional study)			X
Mulheran M, et al., 2001 (Observational, cross-sectional study, with paired control group).			X
McRorie TI, Bosso J, Randolph L, 1989 (Observational, paired case-control study).			X
Pedersen SS, et al., 1987 (Observational, cross-sectional study).			X

Quality of evidence: Class A, high; Class B, moderate; Class C, low or very low.

## Discussion

All studies included in this review report hearing loss in groups of CF patients receiving treatment with ototoxic drugs. However, statistically significant results were observed in five of these studies that performed HFA in patients with cystic fibrosis treated with AGs.^18^, ^23^, ^24^, ^25^, ^26^

In the other four studies of this review, the authors observed that the risk for cochleotoxicity in CF is relatively low, with no significant difference between patient and control groups. These studies suggest carrying out additional research that may include the cumulative effect of drug use.^14^, ^27^, ^29^ These less significant results seem to be related to the short-term follow-up of patients in these studies.

Regarding the applicability of HFA, all studies recommend its use for auditory monitoring as a complementary assessment to pure tone audiometry (PTA) for CF patients.

A systematic review carried out to analyze the national scientific production regarding the clinical application of HFA and its use, concluded that the HFA has been widely used in audiological practice for the early identification of hearing alterations, in the auditory monitoring of subjects exposed to ototoxic drugs or aggressive agents and complementary diagnosis when assessing special populations, as in the case of patients with chronic kidney disease, diabetes and hearing disorders.^17^

Part of the studies included in this study used distortion-product otoacoustic emissions (DPOAE) in the auditory assessment of these patients, showing that this test also contributed to the diagnosis of ototoxicity in children with CF.^18^, ^23^, ^25^, ^26^, ^29^ The advantage of this test is to allow hearing loss detection in children younger than 4 years, who normally cannot be submitted to audiometry, as it requires patient conditioning methods that are not always feasible. As for the limitation of DPOAE use, it usually only detects losses of less than 45 dB HL.

In a study that monitored the hearing of patients exposed to cisplatin, 13 individuals were evaluated through the hearing threshold testing from 250 Hz to 18 kHz, in addition to the transient otoacoustic emissions (TOAE) and distortion product otoacoustic emissions (DPOAE). It was observed that, after the infusion of 120 mg/m^2^ of cisplatin, the auditory thresholds became worse from the frequency of 8 kHz, recorded by the HFA. The overall response and absolute amplitude at frequencies of 1, 2 and 3 kHz of TOAE tend to remain present until the end of treatment, but the same does not occur with the frequency of 4 kHz. The authors concluded that the HFA was the most effective method for early detection of cisplatin ototoxicity.^30^

Four studies included in this review identified more patients with ototoxicity signs through HFA when compared to conventional audiometry.^18^, ^23^, ^24^, ^28^ These studies suggest that HFA should be performed for ototoxicity detection in infants with CF who received treatment with AGs.

In the studies by Mulheran et al. (2001)^14^ and Mulheran et al. (2006)^27^, the decrease in the thresholds at high frequencies is observed in both the control group and in patients with CF. The authors suggested that CF can act as a protective factor against cochleotoxicity because of the faster renal elimination of AGS, reporting that the defect that causes decreased permeability to chloride can damage the transport of AGs into the outer and inner hair cells of the cochlea. Other factors related to hearing loss, such as prematurity and history of intrauterine infection, in addition to AGs use, could contribute to a higher prevalence of hearing loss in the group that did not received AGs.

Two studies in this review suggested that the use of AGs is not the only causal factor for hearing loss in CF.^23^, ^29^ According to Al-Malky et al. (2011)^23^ the occurrence of hearing loss has been associated with high exposure to AGs; however, the high exposure resulted in hearing loss in a minority of patients. According to the authors of this study, genetic analysis may help explain the dichotomy found in response to GAs.

Conrad et al. (2008)^31^ verified that genetic predisposition through mitochondrial mutation (A15555G) makes patients more susceptible to hearing loss caused by AGs, even with smaller doses of the drug. New prospective studies may help to elucidate the cause of hearing loss in CF.

A systematic review that sought to evaluate the role of routine hearing screening for sensorineural hearing loss in children with CF treated with AGs showed that the most frequently used test in the studies was the HFA (6/12), followed by DPOAE (4/12), and only one study included the TOAE (1/12) and auditory brainstem response (1/12).^32^ Other studies using HFA for auditory monitoring in patients during treatment with ototoxic drugs (chemotherapy or renal function conservative agents), reinforce the importance of the test's clinical applicability to prevent permanent hearing loss.^30^, ^33^, ^34^

The study by Martins et al. (2010)^29^ recorded hearing loss in 4% of the sample when considering the mean tritone frequencies of 500, 1000 and 2000 Hz. When using other criteria to define hearing loss, which considered an increase in the auditory threshold in two or more frequencies, including the high ones, hearing loss higher than 25 dB HL was found in 11% of the patients. Since it is known that AGs cause hearing loss initially at high frequencies, which are disregarded in the first criterion, it reinforces the importance of auditory monitoring by HFA. Thus, even if patients have no clinically significant hearing loss, they may benefit from the monitoring that includes high frequencies, when a new treatment with ototoxic medication is started. The variability of the criteria used reinforces the idea that hearing loss can be underdetected when the conventional hearing loss criteria are used.^35^

The AG antibiotic route of administration was observed in the studies of this review, as shown in the following table (Table 3).

**Table 3:** AG antibiotic administration route observed in the studies.

Drug administration route	Number of studies
Intravenous	7
Inhaled	0 (zero)
Both	1
No information on drug administration route	1

The pharmacokinetics of all AGs is quite similar. Due to their polar nature, they are poorly absorbed from the gastrointestinal tract, with less than 1% of the dose being absorbed after oral or rectal administration. The main route of administration is therefore parenteral, with the drug reaching maximum plasma concentration 30–90 min after an intramuscular injection and 30 min after an intravenous injection. Antibiotic therapy is indicated for the treatment of disease exacerbation and for preventive treatment aiming to suppress the bacterial load.^36^

The most recent evidence has justified the use of inhaled antibiotic therapy in patients with chronic infection from *Pseudomonas aeruginosa*, as maintenance or chronic suppression treatment, especially in CF patients. Although the use of inhaled tobramycin is based on randomized controlled trials,^37^ because of its high cost, its use has been reserved for patients who did not tolerate or did not respond to other antibiotics.^38^, ^39^

Among the three studies that evaluated and compared hearing in adults and children with CF receiving AGs,^14^, ^24^, ^27^ two found more hearing alterations in adult patients.^14^, ^24^ McRorie, Bosso and Randolph (1989)^24^ found significant differences in frequencies above 16,000 Hz in patients younger than 20 years and high thresholds at all frequencies tested in patients older than 20 years. In the study by Mulheran (2001),^14^ which evaluated 70 CF patients using AGs, 27 children and 43 adults, it was found that only one of the children had hearing loss attributed to the use of AGs. When the results in adults were analyzed, it was found that 11 patients showed hearing loss attributed to AGs. The more significant results in the group of adult patients may be associated with long-term use of ototoxic medication, suggesting a cumulative risk.

The study carried out by Mulheran et al. (2006)^27^ suggests strong scientific evidence, as it is a randomized controlled trial. This study was carried out with 125 children and 94 adults with CF, aiming to investigate the cumulative risk of ototoxic drug use. It compared a group of patients undergoing treatment with a daily dose of tobramycin to a group treated with three doses of the drug. The audiometry was performed at the start of the treatment, at the end of a 14-day treatment and 6–8 weeks after treatment completion. The hearing thresholds in both treatment groups decreased between 25 and 75 dB HL at the frequencies of 10–16 kHz. Therefore, the loss of hearing threshold in these patients was found with both lower and higher doses of ototoxic medication. Hearing loss was identified by HFA in these patients, but it was not possible to verify differences between the treatment doses, as the study intended.

However, there is strong evidence that HFA can identify early signs of hearing loss or progressive decreases in auditory thresholds, even if they are not exclusively related to the use of ototoxic drugs.

## Conclusion

High-frequency audiometry has been researched and used as a diagnostic and monitoring tool for hearing loss in patients with cystic fibrosis.

This review identified the occurrence of hearing loss at high frequencies in patients with cystic fibrosis who did not have hearing complaints.

It is postulated that high-frequency audiometry may be an early diagnostic method to be recommended for hearing investigation in patients at risk of ototoxicity.

## Conflicts of interest

The authors declare no conflicts of interest.
