# Erratum of “Hearing thresholds at high frequency in patients with cystic fibrosis: a systematic review”^☆^

**DOI:** 10.1016/j.bjorl.2017.08.001

**Published:** 2017-09-11

**Authors:** Debora T.M. Caumo, Lúcia B. Geyer, Adriane R. Teixeira, Sérgio S.M. Barreto

**Affiliations:** a Universidade Federal do Rio Grande do Sul (UFRGS), Faculdade de Medicina (Famed), Saúde da Criança e do Adolescente, Porto Alegre, RS, Brazil; b Hospital de Clínicas de Porto Alegre (HCPA), Serviço de Otorrinolaringologia, Porto Alegre, RS, Brazil; c Universidade Federal do Rio Grande do Sul (UFRGS), Instituto de Psicologia, Departamento de Saúde e Comunicação Humana, Porto Alegre, RS, Brazil; d Universidade Federal do Rio Grande do Sul (UFRGS), Faculdade de Medicina (Famed), Departamento de Medicina Interna, Porto Alegre, RS, Brazil

In the article “Hearing thresholds at high frequency in patients with cystic fibrosis: a systematic review” [Braz J Otorhinolaryngol. 2017;83(4):464–474], where it reads Adriana R. Teixeira, it should read Adriane R. Teixeira.

The online version of the article has already been corrected.

